# Supplementary material for: A Critical View of the Application of the APEX Software (Aqueous Photochemistry of Environmentally-Occurring Xenobiotics) to Predict Photoreaction Kinetics in Surface Freshwaters
Source: Molecules. 2019 Dec 18;25(1):9. doi: 10.3390/molecules25010009 (PMC7017383; doi:10.3390/molecules25010009)
Supplement: Supplementary file 1 [file molecules-25-00009-s001.zip › 3.2.4_gcc-4.4.0.zip!/3.2.4_gcc-4.4.0/3.2.4_gcc-4.4.0/doc/octave/html/interpreter/Predicates-for-Numeric-Objects.html]

Predicates for Numeric Objects - Untitled


Previous: Promotion and Demotion of Data Types,
Up: Numeric Data Types


---

### 4.8 Predicates for Numeric Objects

Since the type of a variable may change during the execution of a
program, it can be necessary to do type checking at run-time. Doing this
also allows you to change the behavior of a function depending on the
type of the input. As an example, this naive implementation of `abs`
returns the absolute value of the input if it is a real number, and the
length of the input if it is a complex number.

```
     function a = abs (x)
       if (isreal (x))
         a = sign (x) .* x;
       elseif (iscomplex (x))
         a = sqrt (real(x).^2 + imag(x).^2);
       endif
     endfunction
```

The following functions are available for determining the type of a
variable.

— Built-in Function: **isnumeric** (x)  
> Return nonzero if x is a numeric object.

— Built-in Function: **isreal** (x)  
> Return true if x is a real-valued numeric object.

— Built-in Function: **isfloat** (x)  
> Return true if x is a floating-point numeric object.

— Built-in Function: **iscomplex** (x)  
> Return true if x is a complex-valued numeric object.

— Built-in Function: **ismatrix** (a)  
> Return 1 if a is a matrix. Otherwise, return 0.

— Function File: **isvector** (a)  
> Return 1 if a is a vector. Otherwise, return 0.
>
>
>
> **See also:** size, rows, columns, length, isscalar, ismatrix.

— Function File: **isscalar** (a)  
> Return 1 if a is a scalar. Otherwise, return 0.
>
>
>
> **See also:** size, rows, columns, length, isscalar, ismatrix.

— Function File: **issquare** (x)  
> If x is a square matrix, then return the dimension of x.
> Otherwise, return 0.
>
>
>
> **See also:** size, rows, columns, length, ismatrix, isscalar, isvector.

— Function File: **issymmetric** (x, tol)  
> If x is symmetric within the tolerance specified by tol,
> then return the dimension of x. Otherwise, return 0. If
> tol is omitted, use a tolerance equal to the machine precision.
> Matrix x is considered symmetric if
> `norm (`x `-` x`.', inf) / norm (`x`, inf) <` tol.
>
>
>
> **See also:** size, rows, columns, length, ismatrix, isscalar, issquare, isvector.

— Function File: **isdefinite** (x, tol)  
> Return 1 if x is symmetric positive definite within the
> tolerance specified by tol or 0 if x is symmetric
> positive semidefinite. Otherwise, return -1. If tol
> is omitted, use a tolerance equal to 100 times the machine precision.
>
>
>
> **See also:** issymmetric.

— Built-in Function: **islogical** (x)  
> Return true if x is a logical object.

— Function File: **isprime** (n)  
> Return true if n is a prime number, false otherwise.
>
> Something like the following is much faster if you need to test a lot
> of small numbers:
>
> ```
>              t = ismember (n, primes (max (n (:))));
> ```
>
> If max(n) is very large, then you should be using special purpose
> factorization code.
>
>
>
> **See also:** primes, factor, gcd, lcm.
